# Chorioamnionitis Induces a Specific Signature of Placental ABC Transporters Associated with an Increase of miR-331-5p in the Human Preterm Placenta

**DOI:** 10.1159/000487100

**Published:** 2018-01-29

**Authors:** Guinever Eustaquio do Imperio, Enrrico Bloise, Mohsen Javam, Phetcharawan Lye, Andrea Constantinof, Caroline Dunk, Fernando Marcos dos Reisf, Stephen James Lye, William Gibb, Tania Maria Ortiga-Carvalho, Stephen Giles Matthews

**Affiliations:** a Departments of Physiology; b Obstetrics and Gynecology and; c Medicine, University of Toronto, Toronto, Ontario Canada; d Laboratory of Translational Endocrinology, Biophysics Institute Carlos Chagas Filho, Federal University of Rio de Janeiro, Rio de Janeiro, Brazil; e Departments of Morphology and; f Obstetrics and Gynecology, Federal University of Minas Gerais, Belo Horizonte, Brazil; g Lunenfeld-Tanenbaum Research Institute, Mount Sinai Hospital, Ontario, Canada; h Departments of Obstetrics & Gynecology and Department of Cellular & Molecular Medicine, University of Ottawa, Ontario, Canada

**Keywords:** Placenta, Chorioamnionitis, Preterm delivery, ABC transporters, P-glycoprotein (P-gp), Breast cancer resistance protein (BCRP)

## Abstract

**Background/Aims:**

The ATP-binding cassette (ABC) transporters mediate drug biodisposition and immunological responses in the placental barrier. *In vitro* infective challenges alter expression of specific placental ABC transporters. We hypothesized that chorioamnionitis induces a distinct pattern of ABC transporter expression.

**Methods:**

Gene expression of 50 ABC transporters was assessed using TaqMan® Human ABC Transporter Array, in preterm human placentas without (PTD; n=6) or with histological chorioamnionitis (PTDC; n=6). Validation was performed using qPCR, immunohistochemistry and Western blot. MicroRNAs known to regulate P-glycoprotein (P-gp) were examined by qPCR.

**Results:**

Up-regulation of *ABCB9, ABCC2* and *ABCF2* mRNA was detected in chorioamnionitis (p<0.05), whereas placental ABCB1 (P-gp; p=0.051) and *ABCG2* (breast cancer resistance protein-BCRP) mRNA levels (p=0.055) approached near significant up-regulation. In most cases, the magnitude of the effect significantly correlated to the severity of inflammation. Upon validation, increased placental *ABCB1* and *ABCG2* mRNA levels (p<0.05) were observed. At the level of immunohistochemistry, while BCRP was increased (p<0.05), P-gp staining intensity was significantly decreased (p<0.05) in PTDC. miR-331-5p, involved in P-gp suppression, was upregulated in PTDC (p<0.01) and correlated to the grade of chorioamnionitis (p<0.01).

**Conclusions:**

Alterations in the expression of ABC transporters will likely lead to modified transport of clinically relevant compounds at the inflamed placenta. A better understanding of the potential role of these transporters in the events surrounding PTD may also enable new strategies to be developed for prevention and treatment of PTD.

## Introduction

Preterm delivery (PTD) is a public health problem, occurring in 5-18% of all pregnancies [1] and it is the leading cause of perinatal mortality and morbidity [2]. Intrauterine infection or chorionamnionitis are responsible for up to 40% of all PTD cases worldwide [3–7]. Important components of the pathophysiology of PTD, such as infection and/or inflammation, have been shown to influence the expression of selected ATP-binding cassette (ABC) transporters [8]. The ABC transporters comprise 50 proteins, subdivided into seven sub-families (ABCA through ABCG), that actively transport a wide array of substrates. Placental ABC transporters exert a critical role regulating steroid transport, immunological responses and drug biodisposition. Importantly, the ABC transporters namely P-glycoprotein (P-gp; encoded by *ABCB1*); breast cancer resistance protein (BCRP; *ABCG2*) and specific multidrug resistance-associated proteins (MRPs; encoded by the *ABCC* subset) are important components of the placental syncytiotrophoblast barrier [8, 9]. They act as “gatekeepers” protecting the fetus against accumulation of potential harmful factors that may be present in the maternal circulation [8].

Acute bacterial infection, modeled by lipopolysaccharide (LPS) exposure, impaired placental P-gp activity resulting in increased fetal drug accumulation in the mouse [10]. LPS insult of first trimester human placental explants decreased the expression of *ABCB1* and *ABCG2* mRNA as well as P-gp and BCRP protein, but had no effect when administered to third trimester placental explants [11], demonstrating that LPS is more likely to disrupt P-gp and BCRP expression during earlier stages of pregnancy. However, little is known as to how infection and/or inflammation impact the expression of these and other placental ABC transporters in cases of chorioamnionitis. Evidence also suggests that selected ABC transporters are key mediators of immunological responses [12] involved in labor [13, 14]. Also, P-gp transports pro-inflammatory cytokines out of cells [15, 16]. Therefore, understanding how infection and inflammation regulate the expression of ABC transporters in the placenta is key to understanding maternal-fetal transfer of steroid hormones, toxins, xenobiotics and immunological factors in pregnancies complicated by chorioamnionitis.

MicroRNAs (miRNAs) are approximately 22 nucleotides in length, single-stranded, non-coding RNAs that are currently being studied as modulators of drug metabolism and disposition via the regulation of nuclear receptors, drug-metabolizing enzymes and drug transporters [17–22]. To date, no study has comprehensively examined the ABC transporter superfamily or involved in the post-transcriptional regulation of these transporters in the human preterm placenta, and how chorioamnionitis modifies their expression. Therefore, we investigated the expression of ABC transporters and related miRNAs in preterm placenta with chorioamnionitis (PTDC) compared to preterm placentas without chorioaminionitis (PTD). We hypothesized that inflammation induces a distinct signature of placental ABC transporters potentially associated with the modulation of specific miRNAs expression in preterm pregnancies and that this signature correlates to the severity of inflammation.

## Materials and Methods

### Sample collection

Placental specimens were obtained from the Research Centre for Women’s and Infants’ Health (RCWH) BioBank (Mount Sinai Hospital, Toronto, CA) with informed consent and in accordance to the policies of Mount Sinai Hospital and the University of Toronto Research Ethic Boards. Placental samples were selected from pregnancies ending in PTD with histologic chorioamnionitis (PTDC; in total n=6) or PTD without histologic chorioamnionitis (PTD; in total n=6). Diagnosis of histologic chorioamnionitis (graded 0-3) was performed by Mount Sinai Hospital staff pathologists based on the presence of polymorphonuclear leukocyte infiltration within the chorionic plate and/or chorioamniotic membranes as previously described [23, 24].

In order to decrease variability, only pregnancies carrying male Caucasian fetuses were selected as an inclusion criteria, since ethnicity [25–28] and gender [29–33] can directly affect inflammatory responses and birth outcome. Exclusion criteria included samples from pregnant women experiencing PTD associated with asthma, cardiovascular diseases, cervical incompetence, diabetes, fetal growth restriction, fetal malformation, hypertension, multiple gestation, preeclampsia, sexually transmitted diseases, thyroid disease and uterine malformations [34]. The clinical profiles of all pregnancies enrolled in the study are presented in Table 1.

**Table 1:** Clinical profile of the placental tissues. Results are presented as mean ± SEM. None of the parameters investigated showed statistical difference between the groups. BMI, Body Mass Index; P, positive; N, negative; U, unknown;V, vaginal; C, Caesarean section with no labor; CL, Caesarean section with labor; GBS, Group B Staphylococcus; WBC, White Blood Cells; S1, Stage1; S2, Stage2; S3, Stage3; HP, Haemorrhage+PPROM (Preterm Premature Rupture of Membranes)

	Preterm Delivery without Chorioamnionitis PTD (N=6)	Preterm Delivery with Chorioamnionitis PTDC (N=6)	P Value
Maternal Characteristics			
Age (years)	27.7 ± 5.6	31.8 ± 4.1	0.56
BMI	22.1 ± 4.8	26.2 ± 4.7	0.65
Ethnicity	Caucasian	Caucasian	
WBC (×10^9^/L)	3.0 - 10.0	3.0 - 10.0	
Fetal Characteristics			
Gestational age (weeks)	31.6 ± 1.9	28.0 ± 1.7	0.20
Mode of Delivery (V:C:CL)	2:1:3	4:2:0	
Neonatal birth weight (g)	1908 ± 554	1185 ± 362	0.30
Neonatal sex	Male	Male	
Pathology Characteristics			
Chorioamnionitis (S1:S2:S3:HP)	-	2:2:1:1	
GBS status (P:N:U)	2:2:2	0:5:1	
Glucocorticoids treatment	yes	yes	

### Total RNA extraction and cDNA synthesis

Total RNA was extracted using the RNeasy Plus Universal Mini kit (QIAGEN, Toronto, ON, Canada). The absorbance ratios 260/280 and 260/230 were used to assess the purity of RNA and they were considered satisfactory only when ranged between 1.8 and 2.0. RNA integrity was determined by calculating the RNA Integrity Number (RIN) by capillary electrophoresis, using the Agilent Bioanalyzer 2100 and RNA 6000 Nano Labchip kit (Agilent Biotechnologies, CA, USA) according to manufacturer’s instructions. Only RNAs with RIN greater to or equal to 7 were used in our study, indicating that the RNA samples had minimal degradation products. 1 ng RNA was reverse transcribed into cDNA using the SuperScript® VILO™ cDNA Synthesis kit (Invitrogen, Grand Island, NY, USA).

### ABC Transporters Low Density Array

Screening of placental ABC transporters mRNA expression was assessed using TaqMan® Human ABC Transporter Array microfluidic cards (TLDA, Applied Biosystems, Foster City, CA, USA) containing assays for 50 human ABC transporter genes in addition to 14 endogenous reference gene controls (catalog number 4378700). A total of 6 TLDA cards were used to quantify mRNA expression from PTD and PTDC placentas (n=6/group). Each card was designed to run 2 distinct samples in triplicate and contained 384 wells and 8 reservoirs in total, i.e. 192 wells per sample (64 assays in triplicate). 400 ng of total RNA reverse transcribed in cDNA was used per sample (100 ng RNA/reservoir) in the TLDA cards. qPCR was performed on the Applied Biosystems ViiA™ 7 qPCR System using the following cycling conditions: 95°C for 20s, followed by 40 cycles of 95°C for 1s and 60°C for 20s. Cycle thresholds (CTs) were assessed using Thermo Fisher Cloud online software (Life Technologies), as the average of each sample assayed in triplicate. ABC transporter mRNA levels were normalized to the geometric mean of the 4 most stable reference genes [tyrosine 3-monooxygenase/tryptophan 5-monooxygenase activation protein, zeta polypeptide *(YWHAZ),* beta-2-microglobulin (B2M), RNA polymerase II *(POLR2A)* and TATA-box binding protein (TBP)] and calculated according to the 2^-MCT^ method [35].

### qPCR

Levelsofinter/eutan *(IL)-8and* YWHAZmRNAwereassessed usingSensiFAST™SYBR®Hi-ROX kit(Bioline, Toronto, ON, Canada) and the CFX 96 Real-Time System C1000 Thermal Cycler (Bio-Rad, Mississauga, ON, Canada), with the following cycling conditions: 95^o^C for 30s and 39 cycles of 95^o^C for 5s and 60^o^C for 5s. *IL-8* and *YWHAZ* primers sequences were F:GCAGCCTTCCTGATTTCTGCAGCT and R:CCTTGGGGTCCAGACAGAGCTCT; and F:ACTTTTGGTACATTGTGGCTTCAA and R:CCGCCAGGACAAACCAGTAT, respectively. For validation of the arrays, *ABCB1* and *ABCG2* mRNA expression, as well as *YWHAZ* expression, was measured using identical probes present in the TLDA card (Hs00184491_m1; Hs00184979_m1 and Hs00237047_m1, respectively) and Taqman® Universal Master Mix II (Applied Biosystems). In this case, the parameters used in the qPCR were: 50°C for 2min, 95°C for 10min followed by 40 cycles of 95°C for 15s and 60°C for 60s. Changes in mRNA expression were calculated according to the 2^-MCT^ method [35].

### miRNA analysis

For miRNAs evaluation, cDNA used to analyze miRNAs of interest were generated using the Taqman® miRNA reverse transcription kit (Applied Biosystems) and Taqman® assays specific for each miRNA *(miR-145:* ID002278; *miR-331-5p:* ID002233; *miR-451:* ID001105; *miR-200c:* ID406985). Expression was measured by qPCR using the Taqman® Universal Master Mix II, no Uracyl-N Glycosylase (Applied Biosystems) in a CFX 380 Real-Time system C 1000 TM Thermal Cycler (Bio-Rad), with the following parameters: 95°C for 10min, followed by 40 cycles of 95°C for 15s and 60°C for 60s. miRNA expression was normalized to the geometric mean of the small nucleolar RNAs *U6* (ID001973), *RNU43* (ID001095) and *RNU44* (ID001094). Changes in miRNA expression were calculated according to the 2^-MCT^ method [35].

### Western Blot

Placental protein was extracted by homogenization in RIPA lysis buffer (1 mol/L Tris-HCL pH 6.8, 2% SDS, 10% glycerol, containing protease and phosphatase inhibitor cocktail; Thermo Scientific Waltham, MA, USA), followed by centrifugation (10, 000g, 10min, 4°C). The supernatant protein (30|ig/well) from each sample was separated by electrophoresis (100V, 1h) using 8% SDS-polyacrylamide gel and transferred to nitrocellulose membranes using the iBlot transfer apparatus (Invitrogen). Membranes were blocked with 5% bovine serum albumin (BSA, Sigma-Aldrich, ON, Canada) in Tris-buffered saline Tween (TBS-T, 1h) for P-gp and BCRP, and with 5% skim milk for B-actin. The membranes were then incubated at 4°C overnight, with the following primary antibodies: anti-rabbit MDR1 (1:1,000 Abcam ab170904, Toronto, ON, Canada), anti-mouse BCRP (1:500 Santa Cruz Biotechnology, 221956, Dallas, TX, USA), and anti-goat B-actin (at the same concentration relative to the respective primary antibody, Santa Cruz Biotechnology) in 5% BSA or skim milk TBS-T solution. Membranes were then washed with PBS and incubated (1h) with horseradish peroxidase conjugated secondary antibody (GE Healthcare Bio-Science, Baie D’Urf, QC, Canada) at concentrations of 1:10, 000 for P-gp or 1:15,000 for BCRP. For B-Actin, anti-goat secondary antibody (Bio-Rad, Mississauga, ON, Canada) linked to horseradish peroxidase (1:10, 000) was used. Subsequently, membranes were washed in PBS and the protein-antibody complexes were detected by incubation with Laminate™ Crescendo Western HRP Substrate (Millipore, Oak Drive, California, USA) for 3min and chemiluminescence was detected under UV using the Versa Doc system (Bio-Rad). Protein band intensity was quantified using ImageJ software (National Institutes of Health, USA) and normalized against B-actin signal, the loading control.

### Immunohistochemistry

Paraffin embedded tissue sections were dewaxed and rehydrated, and the endogenous peroxidase activity was blocked using H_2_O_2_ (0.3%) in methanol (30 min). Antigen retrieval was performed by preheating the sections in sodium citrate (10mM) for 6 min and then for 3 min, leaving them in ice for 20 min after each heating. Sections were incubated in protein blocking solution (Dako, Mississauga, ON, Canada) for 1h, prior to overnight incubation with primary antibodies: anti-mouse MDR-1 (1:500, D-11, Santa Cruz Biotechnology) and anti-mouse BCRP (1:200, BXP-21, Santa Cruz Biotechnology). Mouse IgG1 was used instead of the primary antibody as a non-immune control. Sections were washed in PBS and incubated with the anti-mouse secondary antibody (1h), at the same concentration as the specific primary antibody, prior to being incubated with streptavidin-HRP (1h; Dako) and visualized with peroxidase substrate kit DAB (Dako). Slides were then counterstained with hematoxylin.

Placental P-gp and BCRP scoring of stained sections was performed with adaptations as described previously [36–38]. Briefly, sections were analyzed by three independant researchers blinded to patient grouping. Staining intensity of syncytiotrophoblast, villous core was graded semiquantitatively on a scale of 0-3 arbitrary units, with 0 indicating no detectable staining, 1 = weak, 2 = moderate and 3 = strong. The area of the respective regions with positive staining was evaluated using the following scale: 0 =no detectable staining, 1 =up to 10%, 2=10-50% and 3=more than 50% of staining.

### Statistical Analysis

Statistical analyses were performed using Prism software (GraphPad Software, Inc., San Diego, CA). Data were analyzed using the Kolmogorov-Smirnov normality test followed by Levene’s median test to verify equality of variances. Groups were analysed by unpaired t-test or non-parametric Mann-Whitney test. Data are expressed as mean ± SEM. Non-parametric Spearman correlation was used to correlate the relative quantities of the transporters, *IL-8* and *miR-331-5p* with the degree of chorioamnionitis, obtained from the clinical data. Differences were considered significant when p<0.05.

## Results

### Clinical Data

In the current study, we made every effort to minimize the variability of our analysis, given that both ethnicity and sex may influence the development and intensity of the inflammatory process [39–42]. For this reason, we only selected placental samples from caucasian mothers carrying male fetuses. In addition, there was no significant difference in maternal age (27.7 ± 5.6 vs. 31.8 ± 4.1, p = 0.56), body mass index (BMI, 23.1 ± 4.8 vs. 26.2 ± 4.6, p = 0.65), gestational age at delivery (31.5 ± 1.9 vs. 28 ± 1.7, p = 0.20) or birth weight (1908 ± 554 vs. 1185 ± 362, p = 0.30) between the PTD and PTDC groups (Table 1).

**Fig. 1:**
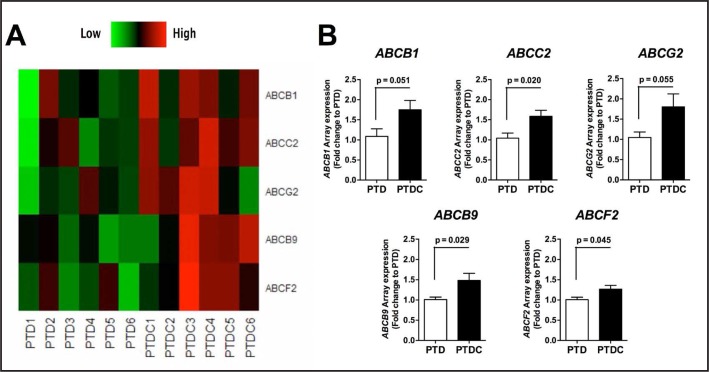
Profile of ATP-binding cassette (ABC) different expressed transporters in the preterm placenta with chorioamnionitis. A) Heatmap derived from the Human ABC Transporters Taqman® Array showing the relative quantities (Rq) of placental ABC transporters in preterm delivery with chorioamnionitis (PTDC) and preterm delivery in the absence of chorioamnionitis (PTD). Higher expression in red, lower in green. B) Fold-change of the transporters, comparing PTD to PTDC. Statistical analysis: unpaired t-test. Data are presented as mean ± SEM (n=6/group).

**Fig. 2:**
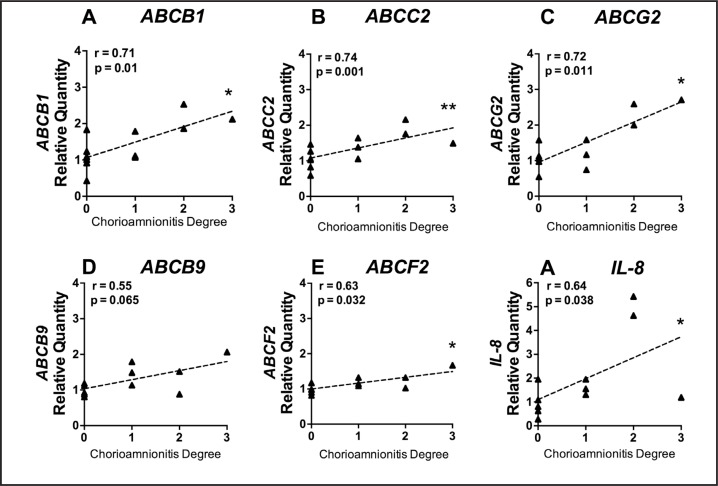
qPCR of selected genes. A) *ABCB1;* B) *ABCG2;* and C) interleukin (IL)-8. Gene expression assessed by qPCR in preterm delivery with chorioamnionitis (PTDC) and preterm delivery in the absence of chorioamnionitis (PTD). Data are presented as fold-change relative to PTD group. Statistical analysis: unpaired t-test for ABCB1 and ABCG2; non-parametric Mann-Whitney test for IL-8. Data are presented as mean ± SEM (n=6/group).

*Gene expression profiling of ABC transporters in preterm placentas with chorioamnionitis* Of the 50 ABC transporters assayed, 48 were detected in the preterm placenta (Table 2]. *ABCG5* and *ABCG8* expression were below the limit of detection. Placental *ABCB9* (p = 0.029], *ABCC2* (p = 0.020) and *ABCF2* (p = 0.045) were significantly up-regulated in the PTDC group (Fig. 1B), whereas placental *ABCB1* (P-gp; p = 0.051) and *ABCG2* (BCRP; p = 0.055) approached near significant up-regulation (Fig. 1A). Further measurement of *ABCB1* and *ABCG2* mRNA by individual qPCR revealed a statistically significant increase of both *ABCB1* (p = 0.027) and *ABCG2* (p = 0.020) in the PTDC compared to PTD (Fig. 2A&B). Thus, chorioamnionitis is associated to an increased expression of 5 placental ABC transporters (Fig. 1&2).

**Table 2:** Fold-change in ABC transporter expression in the presence of chorioamnionitis in the preterm human placenta. *p<0.05. Fold-change is calculated by the ratio between PTDC and PTD relative expression. A fold-change > 1.0 indicates increased expression; fold-change < 1.0 indicates decreased expression; and fold-change = 0 indicates no change in relative expression

ABCA Gene	Fold Change	ABCB Gene	Fold Change	ABCC Gene	Fold Change	ABCD Gene	Fold Change	ABCE Gene	Fold Change
ABCA1	1.01	ABCB1	1.61*	ABCC1	1.23	ABCD1	0.86	ABCE1	1.18
ABCA2	0.96	ABCB2	1.06	ABCC2	1.52*	ABCD2	0.81		
ABCA3	1.17	ABCB3	1.00	ABCC3	0.95	ABCD3	1.35		
ABCA4	2.32	ABCB4	1.64	ABCC4	1.13	ABCD4	1.11		
ABCA5	0.86	ABCB5	0.67	ABCC5	1.21				
ABCA6	0.94	ABCB6	1.35	ABCC6	2.10				
ABCA7	0.81	ABCB7	1.23	ABCC7	0.91	**ABCF**	**Fold**	**ABCG**	**Fold**
ABCA8	0.77	ABCB8	1.11	ABCC8	1.6	**Gene**	**Change**	**Gene**	**Change**
ABCA9	0.83	ABCB9	1.47*	ABCC9	1.41	ABCF1	1.26	ABCG1	1.00
ABCA10	0.62	ABCB10	0.99	ABCC10	1.06	ABCF2	1.26*	ABCG2	1.72*
ABCA11	0.91	ABCB11	1.21	ABCC11	0.60	ABCF3	1.00	ABCG4	0.10
ABCA12	0.99			ABCC12	0.84				
ABCA13	0.82			ABCC13	0.93				

**Fig. 3:**
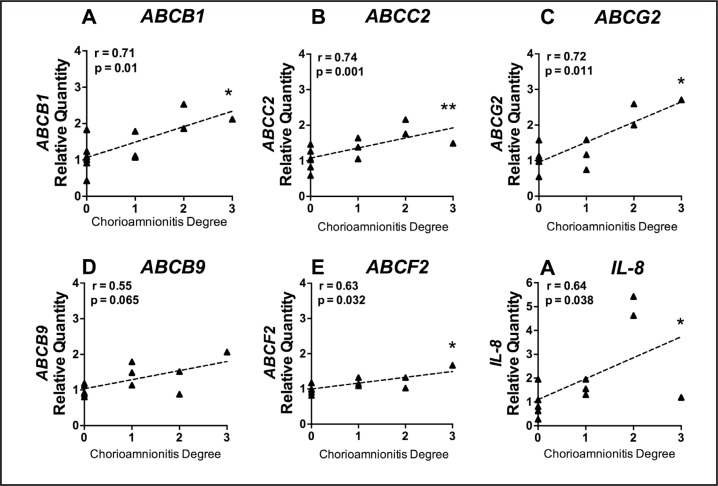
Correlation between mRNA expression of selected transporters and IL-8, and the degree of chorioamnionitis in each placental sample. The degree of inflammation is presented from 0 to 3, with stages 1, 2 and 3 classified as previously described [23]. All preterm delivery placentas without chorioamnionitis (PTD) samples are considered as stage 0. Statistical analysis: non-parametric Spearman correlation (n=6/group).

We next examined the mRNA expression of *IL-8,* a cytokine related to intraamniotic infection [43] (Fig. 2C). *IL-8* mRNA was significantly increased in PTDC compared to PTD (p = 0.030). We also found a significant positive correlation between *IL-8* expression and the severity of chorioamnionitis. Similarly, there was a significant correlation between the severity of chorioamnionitis and the increased expression of *ABCB1, ABCC2, ABCF2* and *ABCG2* (Fig. 3). Of note, *ABCB1* and *ABCG2* exhibited the higher percentage of mRNA increment (61% and 72% respectively) compared to *ABCB9, ABCC2* and *ABCF2* (47%, 52% and 26% respectively Fig. 1B).

### Altered P-gp and BCRP protein in preterm placentas with chorioamnionitis

**Fig. 4:**
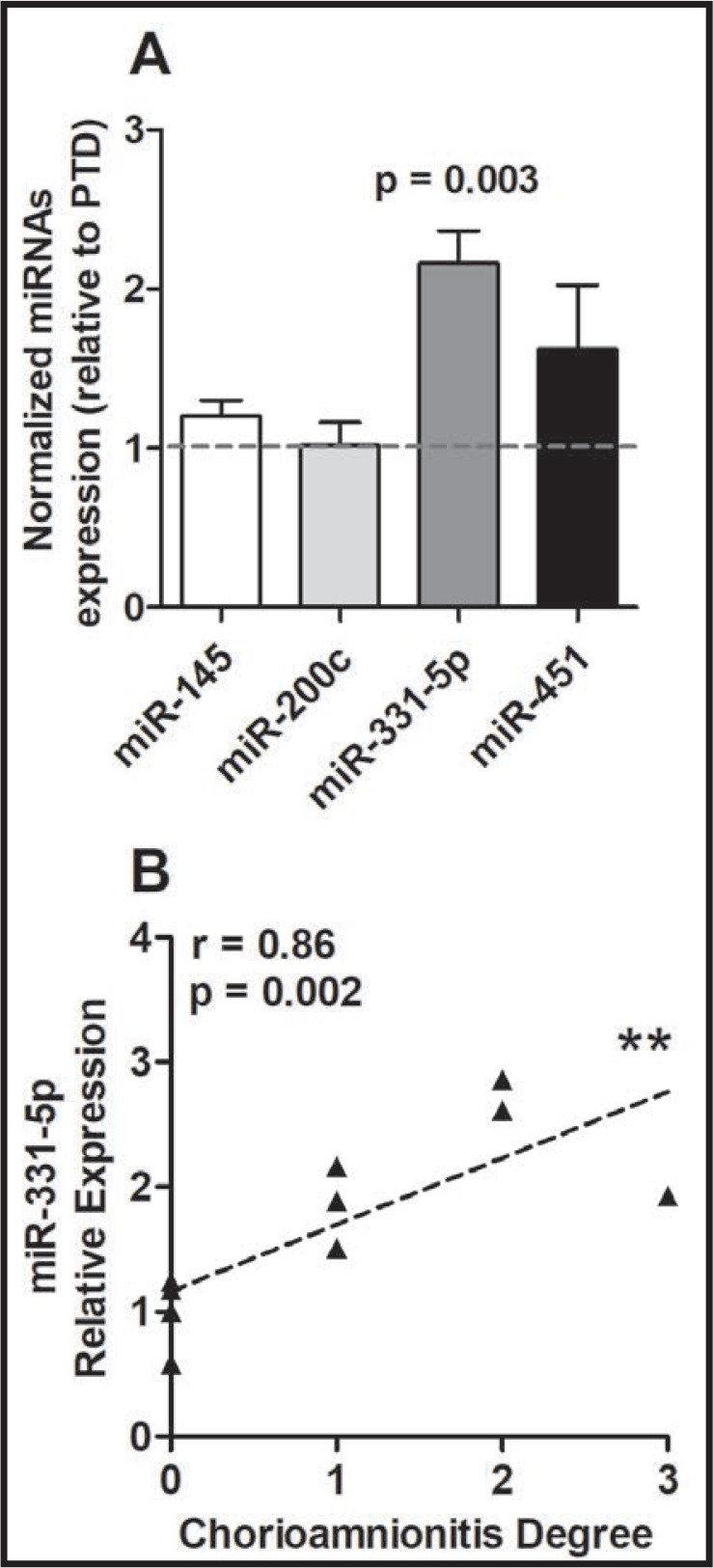
Protein analysis of P-gp and BCRP expression in preterm delivery with (PTDC) and in the absence of chorioamnionitis (PTD). A) Representative P-gp (150 kD), BCRP (72 kDa) and ß-actin (42 kDa) immunoblots followed by placental P-gp and BCRP expression normalized to ß-actin (internal control); B) Representative immunohistochemistry of BCRP; and C) P-gp in PTDC and PTD placentae. Semiquantitative score of the intensity of BCRP staining in the syncytiotrophoblast in PTDC compared to PTD slides. Black arrows indicate BCRP or P-gp staining, predominantly in the cytoplasm and apical membrane of the syncytiotrophoblast. Magnification bars represent 20 urn. Statistical analysis: unpaired t-test. Data are presented as mean ± SEM (n=6/group).

There were no significant differences in P-gp or BCRP (Fig. 4A) levels between groups by Western blot analysis, though there was a strong trend for increased BCRP expression in the PTDC group (Fig. 4A). This trend for an increase was corroborated by immunohistochemistal analysis demonstrating stronger BCRP staining in the syncytiotrophoblast (p = 0.030; Fig. 4B) and villous core (p = 0.009; PTD = 0.14 ± 0.04; PTDC = 0.46 ± 0.08). In contrast, P-gp staining intensity in the syncytiotrophoblast was significantly lower in the PTDC compared to PTD group (p = 0.019, Fig. 4C), with no difference in the villous core intensity (PTD = 0.53 ± 0.11; PTDC = 0.44 ± 0.06).

### Chorioamnionitis-induced changes of miRNAs in human preterm placentas

**Fig. 5:**
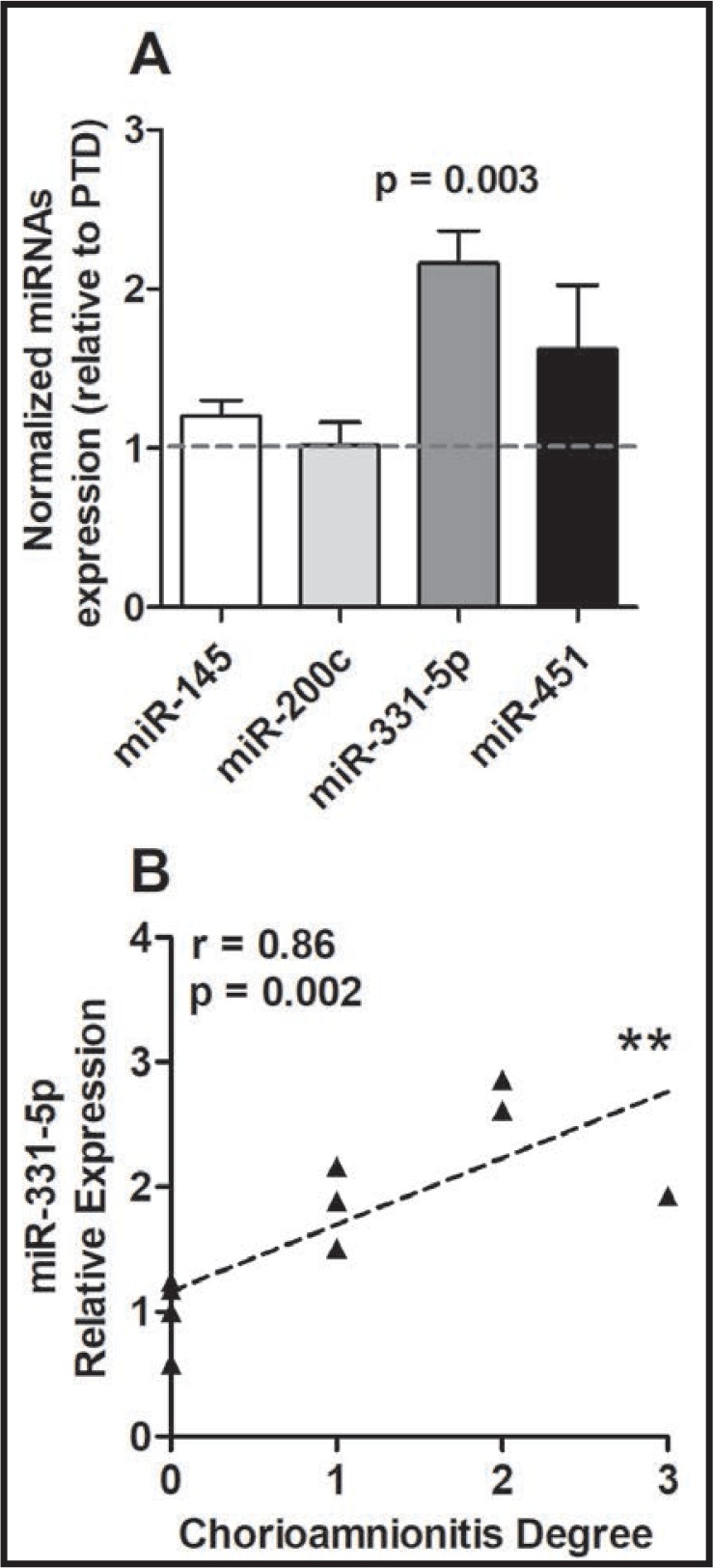
Analysis of miRNAs potentially involved in P-gp regulation by chorioamnionitis in preterm human placenta. A) Expression of selected miRNAs in preterm delivery with (PTDC) and without (PTD) chorioamnionitis placentae; B) Correlation between the miR-331-5p relative expression and the degree of chorioamnionitis of each placentae sample. MiRNA expression was normalized by the geometric mean of the internal controls U6, RNU43 and RNU44. Statistical analysis: A) unpaired t-test; B) non-parametric Spearman correlation. Data are presented as mean ± SEM (n=6/group).

To evaluate the expression of selected miRNAs in preterm placentas with chorioaminionitis, we performed qPCR analysis of miRNAs selected on the basis of their regulatory actions on P-gp *(miR-145, miR-200c, miR-331-5p* and *miR-451)* [44]. Placental expression of *miR-331-5p* (p=0.003) was significantly increased in the PTDC compared to the PTD group (Fig. 5A). Importanty, *miR-331-5p* upregulation was positively correlated (0.77; p = 0.009) to the degree of intrauterine inflammation in preterm human placentas (Fig. 5B).

## Discussion

We have demonstrated that chorioamnionitis alters the placental signature of key ABC transporters, and that these effects correlate to the severity of chorioamnionitis. We have also demonstrated that chorioamnionitis modifies the expression of a specific P-gp regulatory miRNA, the *miR-331-5p.* Such changes have the potential to modify fetal accumulation of a large number of compounds related to obstetric care.

Out of the 50 ABC transporters analyzed by low density array, placental *ABCB9, ABCC2* and *ABCF2* were significantly up-regulated in the PTDC group, whereas the near significant up-regulation observed in *ABCB1* and *ABCG2* was posteriorly validated by individual qPCR, demonstrating that this technique is more sensitive to detect significant changes in gene expression than the low density array, possibly due to the amount of RNA transcribed into cDNA used in the separate reactions, ie. 2 ng of for the arrays, compared to 10 ng for the individual qPCR. Importantly, these changes were directly related to the degree of tissue inflammatory response, as demonstrated by the compelling positive correlation between the relative expression of four out of five up-regulated ABC transporters and the severity of chorioamnionitis in each placental sample.

Since *ABCB1* and *ABCG2* exhibited the largest increase in expression and the strongest correlation with the intrauterine inflammation, and because they are the most well-characterized placental ABC transporters, we further investigated whether these changes extended to the level of protein. The increase in *ABCG2* mRNA in PTDC placentas was mirrored by a trend towards an increase in BCRP protein and a significant increase of BCRP immunostaining in the syncytiotrophoblast and villous core. We have previously demonstrated that LPS treatment, a common model of gram-negative bacterial infection, impairs *ABCG2* and BCRP expression in first trimester human placental explants, but had no effect when administered to third trimester placental explants [11]. *ABCG2* and BCRP are time-depedently expressed in the placenta [1, 37], and are modulated by infection and inflammation in a gestational-age dependent manner [9]. Our previous studies were undertaken using short-term *in vitro* models of infection, whereas the current results were obtained from placentas following preterm delivery in the presence or absence of chorioamnionitis. Thus, this represents quite a different situation from the *in vitro* studies, and this may account for the differences in the ABCG2/BCRP responses that we observed. In the present study, there is likely considerable etiological heterogeneity given the multiple possible causes of the inflammatory process, i.e., the type of pathogen involved, if it is a monomicrobial or polymicrobial infection or indeed, non-infective (i.e. sterile chorioamnionitis) [24, 45–47]. Future studies should examine how different infective agents impact the expression of ABC transporters. In the present study, we were unable to trace-back the causes of chorioamnionitis because the rate of microbiologically-proven intra-amniotic infection is still extremely low [24].

BCRP is highly expressed in the apical membrane of the syncytiotrophoblast [8, 48–50], and the up-regulation may promote increased efflux of BCRP substrates. Therefore, third-trimester chorioamnionitis has the potential to decrease accumulation of BCRP endogenous and exogenous substrates in the fetal compartment including folate, antibiotics and antiretrovirals.

While no change in P-gp was detected by Western blot, immunohistochemistry revealed decreased staining intensity for P-gp in the syncytiotrophoblast of PTDC group. Despite decreased P-gp immunostaining in the syncytiotrophoblast, we observed increased *ABCB1* mRNA expression. This latter result is consistent with a previous study demonstrating that chorioamnionitis increased *ABCB1* mRNA in the human placenta [51]. This disconnect between mRNA and protein has been previously described [49, 52], and may indicate post-transcriptional influences of inflammation. A number of miRNAs have been shown to regulate P-gp levels in cancer cells. miR-331-5p decreased P-gp protein expression in a luciferase reporter assay [53]. In the present study, we discovered that miR-331-5p is upregulated by placental chorioamnionitis and that there was correlation between this upregulation and the degree of placental inflammation. It is possible that while chorioamnionitis increases *ABCB1* mRNA levels a simultaneous increase in miR-331-5p blocks P-gp protein production. Clearly further studies are warranted to investigate this novel relationship in non-malignant tissue.

A reduction of P-gp protein expression following infection and inflammation is consistent with previous studies. We have previously demonstrated that LPS treatment down-regulated P-gp and *ABCB1* expression in first trimester human placental explants, while in third trimester placentas, *ABCB1* expression was down-regulated by polyinosinic:polycytidylic acid (polyI:C) treatment (which models viral infection) but not by LPS [11]. A decrease in P-gp protein expression in PTDC may be associated with fetal accumulation of P-gp substrates, such as endogenous and synthetic glucocorticoids, estrogens and pro-inflammatory compounds. This possibility is supported by the evidence that LPS and polyI:C inhibited P-gp activity and increased fetal drug exposure in pregnant mice [8, 54]. P-gp also transports important classes of drugs such as angiotensin receptor blockers, antibiotics, antiepileptics, antihistamines, antiretrovirals, NSAIDs and statins^1^. In this context, these common medications might accumulate in the fetal compartment to a greater extent in the preterm placenta with chorioamnionitis than in PTD pregnancies without chorioamnionitis, leading to increased fetal drug exposure.

P-gp transports pro-inflammatory cytokines, including IL-2, Interferon (INF)Y, tumor necrosis factor (TNF)-a and chemokines, such as chemokine (C-C motif) ligand 2 (CCL2), into the extracellular space, hence exerting crucial role in regulation of inflammatory response [15, 16]. Simultaneously, pro-inflammatory mediators as TNF-a and IL-1ß down-regulate *ABCB1* mRNA and P-gp levels in human primary placental trophoblast cells [55, 56], which in circumstances as chorioamnionitis, will likely lead to a regulatory loop consisting of P-gp suppression and progressive accumulation of inflammatory factors in the intrauterine environment. Considering that IL-6, IL-1ß e TNFa are key regulators of preterm induction in women [57, 58], the down-regulation of placental P-gp expression induced by pro-inflammatory cytokines may contribute to the induction of preterm labor in this study. In parallel, pharmacological inhibition or molecular silencing of BCRP activity in trophoblast cells led to increased susceptibility to stress-induced apoptosis, a process mediated by TNFa and INFY [56]. Thus, in the context of chorioamnionitis the increase of placental BCRP expression may be associated with enhanced placental and fetal protection against the damage induced by the inflammatory process.

*ABCB9, ABCC2* and *ABCF2* were also up-regulated by chorioamnionitis though very little is known concerning the potential function of these transporters in the placenta. *ABCB9* is a lysosomal/endoplasmic reticulum transporter previously shown to be present in the testis, spinal cord and the brain [59, 60]. A role for *ABCB9* has been proposed at the blood-testes barrier [59] and in T lymphocytes in response to infection [61]. However, its localization and function in the placental barrier, as well as its involvement to chorioamnionitis is yet to be determined. *ABCC2* (MRP2) has been shown to be expressed in the apical membrane of the syncytiotrophoblast [8] and it has important physiological and pharmacological substrates, including hormones, antiretrovirals and opioids [8, 62–64]. The *ABCF2* transporter has been shown to be localized to the cytoplasm and is known to be involved in breast and ovary carcinogenesis [65–67]. *ABCF2* is down-regulated during chronic inflammation of the gastrointestinal tract and up-regulated by TNF-a treatment of HT29 cells [68]. Further studies investigating localization, function and relation to inflammatory processes in the placenta are warranted.

## Conclusion

Inflammation results in a specific signature of ABC transporter expression in the human preterm placenta. Given that ABC transporters extrude a range of specific substrates, alterations in their expression will likely lead to modified transport of clinically relevant compounds as well as physiological factors. A better understanding of the potential role of these transporters in the events surrounding PTD may also enable new strategies to be developed for prevention and treatment of PTD.

## Acknowledgements

We would like to thank Jeremy P. Landry for helping with the semi quantitative scoring and Alisa Kostaki for the basic support in this research.

Conception and design: GEI, EB, WG, TMOG, SGM. Acquisition, analysis and interpretation of data: GEI, EB, MJ, PL, AC, CD. Drafting the article and revising it for important intellectual content: GEI, EB, FMR, WG, SJL, TMOG, SGM. Final approval of the version to be published: GEI, EB, MJ, PL, AC, CD, FMR, WG, SJL, TMOG, SGM.

This study was funded by Bill & Melinda Gates Foundation (MCTI/CNPq/MS/SCTIE/Decit/Bill e Melinda Gates 05/2013], Canadian Institutes for Health Research (SGM; MOP-57746), Conselho Nacional de Desenvolvimento Científico e Tecnológico (CNPq, 304667/2016-1, 422441/2016-3, 303734/2012-4), Coordenacáo de Aperfeicoamento Pessoal de Nivel Superior (CAPES) and Fundacáo de Amparo á Pesquisa do Estado do Rio de Janeiro (FAPERJ, CNE 2015/E26/203.190/2015).

## Disclosure Statement

None declared.
